# Treatment of Early Allergic and Late Inflammatory Symptoms of Allergic Rhinitis with *Petasites Hybridus* Leaf Extract (Ze 339): Results of a Noninterventional Observational Study in Switzerland

**DOI:** 10.3390/ph14030180

**Published:** 2021-02-24

**Authors:** Maren Blosa, Julia Uricher, Sabine Nebel, Catherine Zahner, Veronika Butterweck, Jürgen Drewe

**Affiliations:** Medical Research, Max Zeller Söhne AG, Seeblickstrasse 4, 8590 Romanshorn, Switzerland; maren.blosa@zellerag.ch (M.B.); Julia.uricher@zellerag.ch (J.U.); sa.nebel@bluewin.ch (S.N.); zahner-daniel@bluewin.ch (C.Z.)

**Keywords:** *Petasites* *hybridus*, butterbur, Ze 339, noninterventional study, observational study, allergic rhinitis, inflammatory

## Abstract

The primary objective of this noninterventional, observational study was to assess the effectiveness of the *Petasites hybridus* leaf extract (Ze 339) on early allergic and late inflammatory symptoms of allergic rhinitis in Swiss outpatients. This study was conducted by general practitioners and allergologists. Data from 226 patients were collected during three documented visits. The intermediate visit was ideally made 2–4 weeks after the baseline visit, followed by the final visit approximately 2–4 months later. The mean study duration was 63 days, with 75% of patients being treated for at least 4 weeks. Of the patients, 58.5% started with Ze 339 monotherapy, and 41.5% received other antiallergic and/or sympathomimetic drugs. In both groups, the allergic total symptom score and the inflammatory total symptom scores were significantly (*p* < 0.001) reduced, and the scores for quality of life were improved. Both physicians and patients were very satisfied with the treatment and the concept of therapy, not only for short-term (seasonal) therapy but also for long-term therapy. The tolerability was good: only three mild gastrointestinal adverse events occurred. In summary, the effectiveness of *P. hybridus* leaf extract Ze 339 for the treatment of early allergic and late inflammatory symptoms of allergic rhinitis could be confirmed.

## 1. Introduction

Allergic rhinitis is an inflammatory disorder of the mucosa in the upper airways with the infiltration of inflammatory cells such as neutrophils, eosinophils, basophils and mast cells [1]. It occurs in two subtypes: seasonal allergic rhinitis (SAR) and perennial allergic rhinitis (PAR) [2]. The pathophysiologies of SAR and PAR are very similar with respect to the chemical mediators produced and end-organ manifestation. Differences between SAR and PAR are primarily due to the responsible allergens and the duration of the disease. The immune response involves the interaction of the allergens with specific IgE antibodies bound to high-affinity receptors on the surface of mast cells and basophils in the nasal mucosa [3]. This induces degranulation of these cells, resulting in the release of mediators, which are responsible for a cascade of symptoms. The early symptoms of SAR, e.g., sneezing and rhinorrhea, are mainly due to the rapid release of histamine. Other mediators, such as prostaglandins, leukotrienes and interleukins, are mainly associated with the late-phase responses, which predominantly cause nasal obstruction (congestion) due to allergic inflammation. Nasal obstruction is the most disturbing symptom of AR and is correlated with higher values of IL-4, IL-5 and IL-8 and seems to be closely related to reduced nasal airflow [4]. Allergic rhinitis affects 10–40% of the population and 15–25% of children and young adults. It leads to an impairment of their quality of life, negatively affects school performance and increases work absenteeism due to illness. The direct and indirect costs caused by AR are considerable, so accepted and effective treatments are of high relevance [5,6]. There is considerable comorbidity with asthma: about 60% of asthmatic patients suffer from rhinitis and about 20–30% of patients with AR develop asthma [7].

*Petasitis hybridus* (L.) P.G. Gaertn., B. Mey., & Scherb., commonly known as butterbur, is an herbaceous perennial plant in the family Asteraceae, which is native to Europe and northern Asia. Extracts prepared from rhizomes or leaves of *P. hybridus* have a long history in traditional medicine as anti-inflammatory and spasmolytic drugs for the treatment of various diseases. Biologically active compounds are the sesquiterpenes petasin, isopetasin and neopetasin, which occur in rhizomes, roots and leaves. [8]. Furthermore, *P. hybridus* is well known to contain pyrrolizidine alkaloids (PAs), for which hepatotoxic [9], carcinogenic and mutagenic properties have been reported [10,11]. The PA level depends on which part of the plant is used for extraction: with higher levels occurring in the rhizomes (especially young and fast-growing rhizomes) than in the leaves [12]. Thus, the leaves of *P. hybridus* are more favorable for therapeutic use. Raw, unprocessed *P. hybridus* should not be used long-term due to the potential of PA hepatotoxicity [13]. To reduce the risk of potential hepatotoxic, carcinogenic and mutagenic effects, special extracts devoid of PAs were developed by using sub- and supercritical carbon dioxide extraction. There are several butterbur products available on the market. However, the use should be limited to commercial products that are virtually free of PAs [13].

*P. hybridus* extract Ze 339 is a proprietary CO_2_ extract prepared from the leaves of the plant. The extract Ze 339 is chemically well characterized and is standardized to 8 mg total petasins. The extract Ze 339 is approved in Switzerland and other countries for the symptomatic treatment of allergic rhinitis (hay fever) and related symptoms in the eyes, nose and throat. The efficacy of Ze 339 has been confirmed in several clinical trials [14,15]. Pharmacological studies have shown that Ze 339 and its pharmacologically active constituents, the petasins, possess clear anti-inflammatory effects, which can be mainly explained by the inhibition of synthesis and secretion of various cytokines and leukotrienes [16]. In particular, Ze 339 inhibited Cys-LT and LTB4 synthesis in human macrophages, which had been stimulated with platelet-activating factor (PAF). Further, Ze 339 blocked PAF- as well as the complement peptide C5a-mediated Cys-LT synthesis in eosinophils and LTB4 synthesis in neutrophils. The effects of the positive comparator zileuton, an orally active inhibitor of LT synthesis, were similar to Ze 339 in human eosinophils and neutrophils [17]. The effects of IL-4, IL-6 TNF-α were diminished in both nasal fluids and inflammatory cells. Furthermore, levels of LTB4 and Cys-LT were reduced in nasal fluids and inflammatory cells, but not in peripheral blood leukocytes, suggesting that Ze 339 reacts at the site of inflammation and does not cause systemic immunosuppression [18,19].

In human nasal epithelial cells, Ze 339 mediated changes in proinflammatory mediators, reduced the chemotaxis of neutrophils and had an inhibitory effect on the Janus kinase (JAK) signal transducer and activator of transcription proteins (STAT) signaling pathway [19]. In a clinical mode-of-action study in subjects with AR, Ze 339 significantly reduced interleukin-8 and leukotriene B4 levels in nasal secretions. Ze 339 also showed better efficacy in alleviating nasal obstruction symptoms after unilateral nasal allergen provocation than either desloratadine or placebo and inhibited critical components of the chemokine network [20]. In two randomized clinical trials, the effect of Ze 339 in patients with AR was confirmed by comparison to placebo [21] or the antihistamine fexofenadine [15]. In another study, Ze 339 showed similar tolerability to the antihistamine cetirizine in the same patients. The results from controlled clinical efficacy and safety trials have been supported by several postmarketing studies [22].

The aim of this noninterventional, observational study was to assess the effectiveness and safety of Ze 339 on early allergic and late inflammatory symptoms of AR under the conditions of daily general practice of Swiss physicians.

## 2. Results

### 2.1. Participants, Study Flow and Demographics

**Figure 1:**
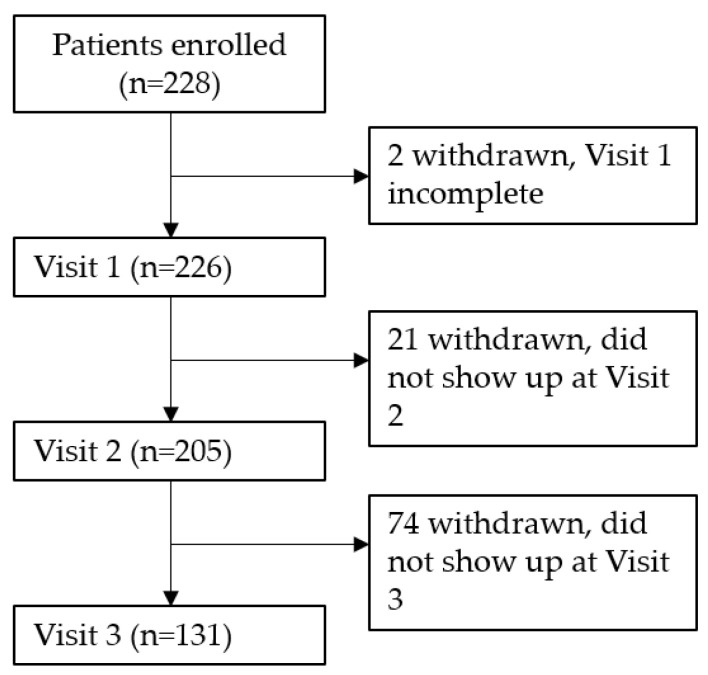
Consort flow chart. Visit 1: baseline visit; Visit 2: intermediate visit; Visit 3: final visit.

In total, 226 patients were included in the study (Figure 1). Of these, 136 (60.2%) patients were female and 90 (39.8%) patients were male. The mean age was 37.3 ± 17.3 (SD) years (Table 1). The majority of the patients (41.6%) were between 36 and 60 years of age (see Table 1).

### 2.2. Allergic History of Participants

**Figure 2:**
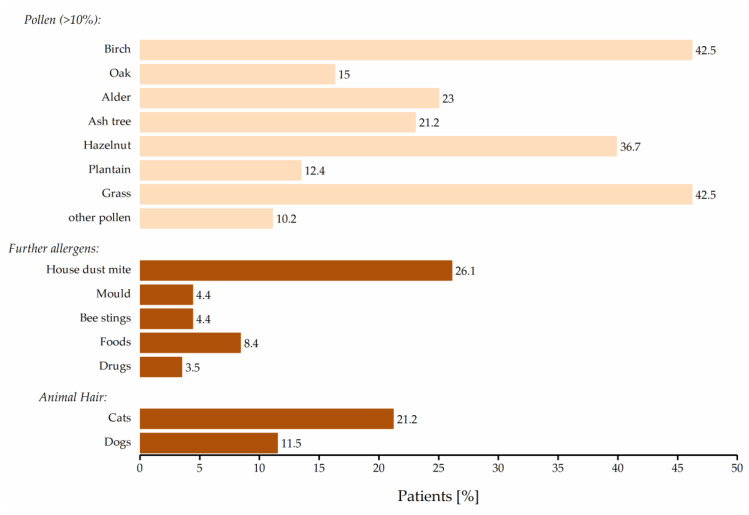
Individual allergens leading to allergic rhinitis symptoms (*n* = 226).

Based on the allergens to which the patients reacted with allergic and inflammatory symptoms (Figure 2) and the time of exposure, participants were categorized either as patients with seasonal rhinitis or perennial rhinitis or combined AR. This differentiation is generally important because those patients with perennial AR also often have a worse quality of life [23].

**Figure 3:**
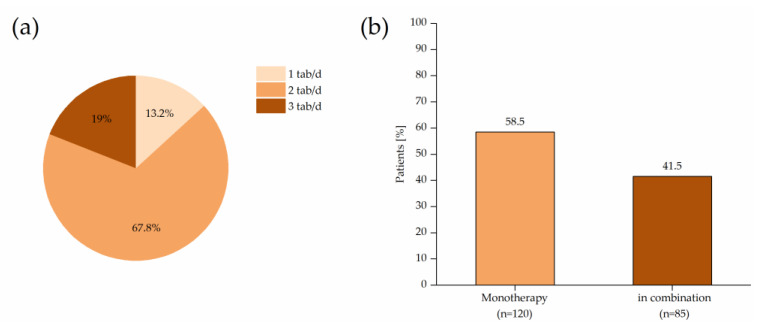
Treatments of patients: (**a**) Treatment regime from the baseline visit (Visit 1). From 226 patients, 28 patients started with 1 tablet daily, 151 patients with 2 tablets daily and 47 with patients 3 tablets daily. (**b**) Frequency of concomitant medication at the beginning of the study (*n* = 205). Eighty-five patients received concomitant drugs.

The majority of patients suffered from seasonal allergic rhinitis, which was often combined with perennial allergic rhinitis (Table 2, Figure 3).

### 2.3. Actual Treatment of Allergic Symptoms and its Modalities

In approximately two-thirds of the cases (*n* = 139, 67.8%), AR was treated with two tablets of Ze 339 daily beginning at Visit 1. Less frequently, three tablets, (*n* = 39, 19.0%) or one tablet (*n* = 27, 13.2%), were administered per day. The daily dose was changed in only 17 patients; six patients increased the daily dosage, and 11 patients reduced their daily dosage. The reasons for this were not documented. 

At the beginning of the study, approximately 40% of the patients suffered from further symptoms of atopic disease such as (rhino-) sinusitis (23.9%), (allergic) asthma (18.3%) and atopic dermatitis (12.2%). A majority of the patients included took Ze 339 as monotherapy (*n* = 120, 58.5%). A slightly smaller population received additional medication for AR or other symptoms of atopic disease (*n* = 85, 41.5%). These patients were additionally treated with antihistamines (55.7%), glucocorticoids (26.8%), sympathomimetics (8.4%), leukotriene antagonists (0.8%) or combinations of these (4.4%). No concomitant drug or supplement containing butterbur was identified.

### 2.4. Treatment-Specific Effectiveness

**Figure 4:**
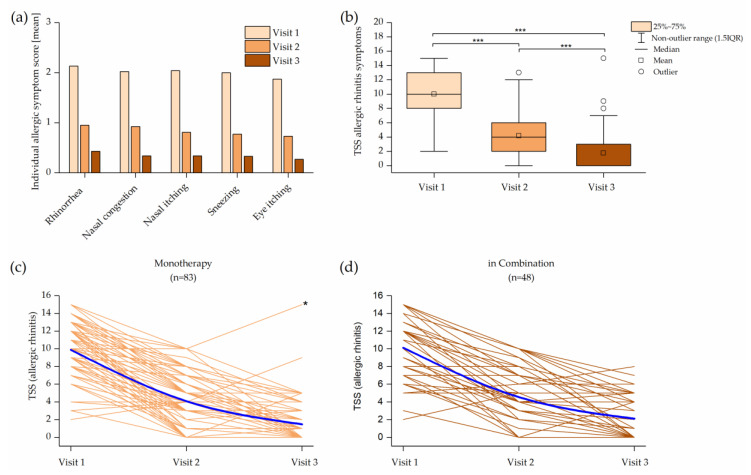
Effect of treatment over the treatment duration: (**a**) individual allergic symptoms for all symptom scores were mutually significant between the visits (*p* < 0.001); (**b**) box plot of the allergic total symptom score (TSS) (*** = *p* < 0.001); (**c**) time course of the individual allergic TSS values for subjects with Ze 339 monotherapy. Patients receiving monotherapy showed a significantly lower symptom score (*p* = 0.023) and lower effect variability (*p* = 0.001) at the end of treatment compared to those receiving concomitant medication. (**d**) Time course of the individual allergic TSS for subjects with concomitant therapy. Blue lines indicate the mean of the TSS. * Patient suffered from chronic respiratory infection.

The effectiveness was assessed by changes in the total symptom score (TSS) from baseline (Visit 1) until the end of the treatment. The TSS of all symptoms at Visit 1 was 17.3 ± 8.2 (SD) and reduced significantly during Ze 339 therapy (*p* < 0.001) (Figure 4a,b). There were no significant differences between patients with or without comedication in the baseline TSS. 

For further analyses, symptom scores were subdivided into allergic TSS, inflammatory TSS and quality of life TSS, where the TSS for each subgroup represents the sum of the individual symptom scores. 

The initial average symptom score of all AR symptoms (rhinorrhea (2.13), nasal congestion (2.02), nasal itching (2.04), sneezing (2.0) and eye itching (1.87)) was categorized as mild to moderate and resulted in a TSS of 9.97. All symptoms improved significantly from Visit 1 to Visit 2 (*p* < 0.001) and again to the final Visit 3 (*p* < 0.001) (Figure 4a,b). Patients receiving monotherapy showed a significantly lower symptom score (*p* = 0.023) and lower effect variability (*p* = 0.001) at the end of treatment compared with those receiving concomitant medication (Figure 4c,d). 

**Figure 5:**
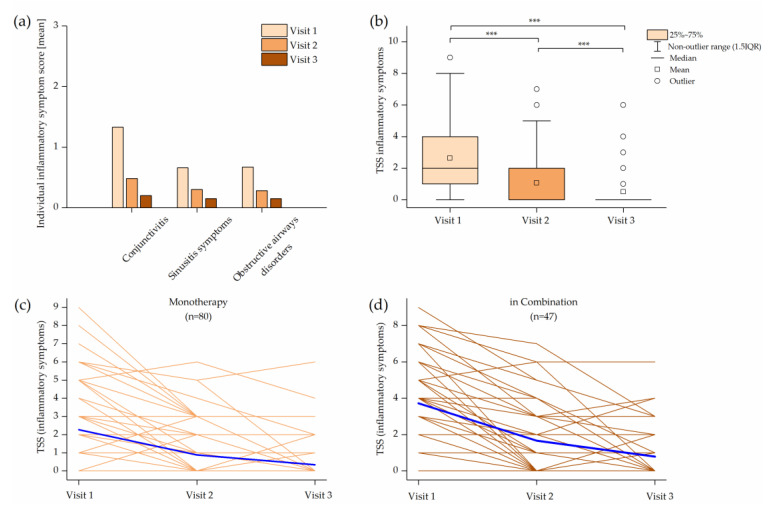
Effect of treatment over the treatment duration: (**a**) individual inflammatory symptoms for all symptom scores were mutually significant between the visits (*p* < 0.01); (**b**) box plot of the inflammatory total symptom score (TSS); (**c**) time course of the individual inflammatory TSS values for subjects with Ze 339 monotherapy; (**d**) time course of the individual inflammatory TSS for subjects with concomitant therapy. Blue lines indicate the mean of the TSS.

The initial average severity of individual inflammatory symptoms (conjunctivitis (1.33), sinusitis symptoms (0.66) and obstructive airways disorders (0.67)) was assessed as absent or mild and declined significantly during the therapy with Ze 339 (*p* < 0.01). The TSS of inflammatory symptoms at Visit 1 was 2.8 and declined significantly to 1.16 at Visit 2 and 0.5 at Visit 3. The reduction in TSS was mutually statistically significant between all visits (*p* < 0.001) (Figure 5a,b). Patients with Ze 339 monotherapy and combination therapy experienced an improvement in inflammatory symptoms (Figure 5c,d). 

### 2.5. Duration of Treatment

**Figure 6:**
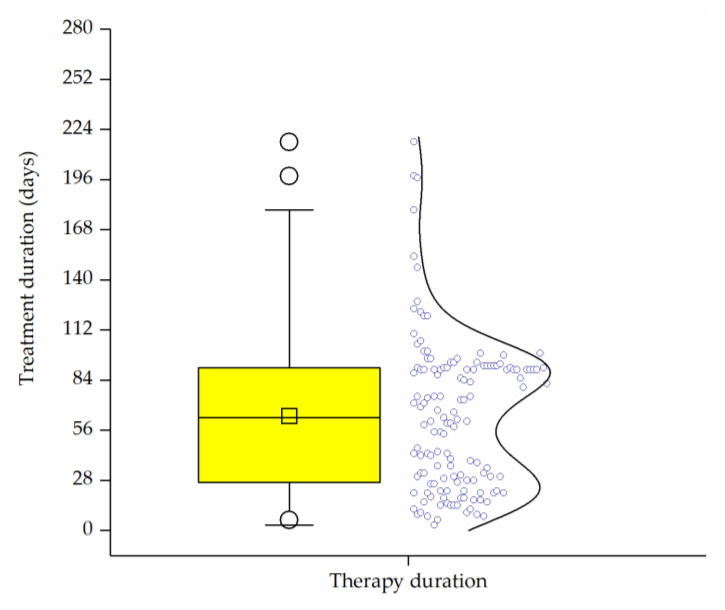
Box plot of treatment duration (days). Individual values are represented by a bimodal distribution density curve (*n* = 139). Data show that approximately 75% of the patients were treated for longer than 28 days.

A minimum treatment duration was not specified and was decided by the physician or patient. The therapy duration was calculated from the start to the end of the study or as indicated by the physician at the last visit (*n* = 139). The patients were treated for a period of 3 days up to 217 days. The mean therapy duration was 63 days and 75% of the patients were treated for at least 4 weeks (Figure 6). Additionally, 67 patients decided to continue the therapy beyond the end of the study, and 20 patients used Ze 339 as a reserve drug after therapy termination. 

### 2.6. Impact on Quality of Life

**Figure 7:**
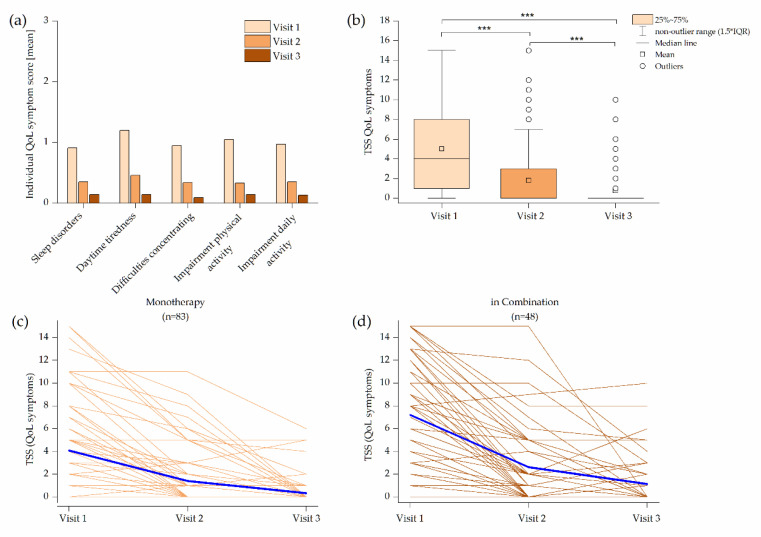
Impact on quality of life (QoL) items: (**a**) individual QoL items between visits; (b) box plot of the QoL total symptom score (TSS); (**c**) time course of the individual QoL TSS values for subjects with Ze 339 monotherapy; (**d**) time course of the individual QoL TSS for subjects with concomitant therapy. Blue lines indicate the mean of the TSS.

The impact of AR on daily life was assessed by five quality of life (QoL) items. The average score of these items (sleeping disorders (0.91), tiredness over the day (1.2), concentration difficulties (0.95), impairment of sportive activities (1.05) and impairment of daily activities (0.97)) improved mutually significantly under Ze 339 treatment (*p* < 0.001). The TSS of QoL items ameliorated from 5.23 at Visit 1 to 1.85 at Visit 2 and 0.63 at Visit 3 (*p* < 0.001) (Figure 7a,b). Patients with Ze 339 monotherapy and combination therapy experienced an improvement in QoL symptoms (Figure 7c,d). 

### 2.7. Effectiveness on Other Allergic and Atopic Symptoms

**Figure 8:**
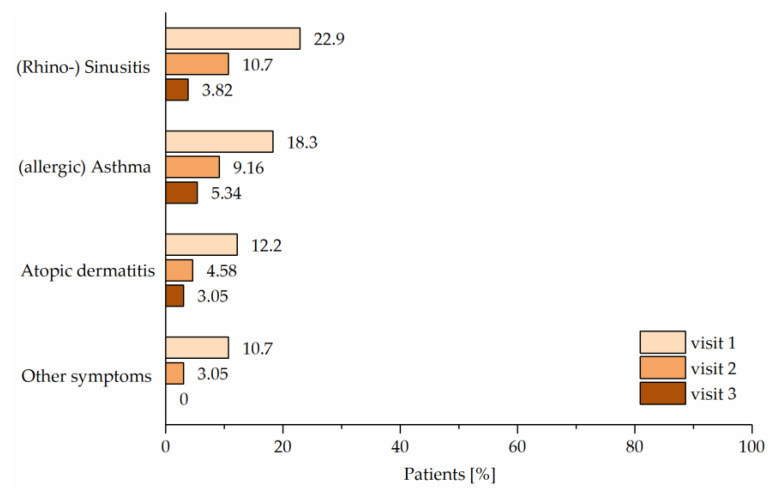
Other symptoms of atopic disease; comparison between the baseline visit (Visit 1) and the final visit (Visit 3) was statistically significant for (rhino-) sinusitis (*p* < 0.003), (allergic) asthma (*p* < 0.05) and atopic dermatitis (*p* < 0.03).

Besides the reduction of AR, patients with atopic comorbidities also seemed to benefit from Ze 339 therapy. In total, the number of patients without other symptoms of atopic diseases increased significantly from Visit 1 (59.5%) to Visit 2 (78.6%) and further to Visit 3 (88.5%). In addition, the number of patients suffering from other symptoms of atopic diseases declined continuously from Visit 1 to Visit 3. (Rhino-) sinusitis (*p* < 0.001), (allergic) asthma (*p* < 0.001) and atopic dermatitis (*p* < 0.003) subsided significantly under Ze 339 therapy (Figure 8).

### 2.8. Tolerability

In general, physicians were very satisfied with the onset of action (2.3), overall effectiveness (2.4), safety (2.9) and compliance (2.7). The antiallergic (2.4) and anti-inflammatory effects (2.3) of Ze 339 were convincing. Furthermore, 85.4% (Visit 2) and 94% (Visit 3) of patients evaluated the concept of therapy as successful and were especially satisfied with the treatment’s onset of action. A comparison of satisfaction between subgroups (monotherapy versus comedication) revealed no significant differences, except for QoL improvement. Patients with Ze 339 monotherapy were significantly more satisfied (*p* < 0.01) than patients with comedication.

The treatment with Ze 339 was well tolerated: just three patients experienced four adverse events: All adverse events were nonserious and were mainly related to the gastrointestinal system (one patient experienced nausea, one patient nausea and malaise and one patient abdominal pain). No treatment was required. The adverse events that occurred were all previously known and had already been included in the summary of product characteristics.

## 3. Discussion

The onset of AR is common in childhood, where it is mainly diagnosed by a physician. A small number of patients improve during adolescence, but symptoms often reoccur in early adulthood [24]. AR is a chronic disease with significant remissions and relapses. Therefore, a high percentage of patients are affected over a lifelong period and are well aware of their symptoms [1]. AR symptoms can affect the nose, eyes and ears. Commonly reported symptoms are nasal itching, nasal congestion, runny nose, sneezing, eye itching, burning and watery eyes [25].

Besides the avoidance of allergens and long-lasting immunotherapy, the use of oral and intranasal medication for AR symptoms is widespread. For the mild form of AR, antihistamines, sympathomimetics or leukotriene antagonists are considered the medication of choice [26]. Intranasal glucocorticoids constitute the most effective treatment of the medium-severe form of AR. In addition, oral or intranasal antihistamines and sympathomimetics or leukotriene antagonists should be considered [27]. Treatment with intranasal chromones may be of benefit for some patients due to the mild side effects, but it has only limited efficacy. Locally applied antihistamines are a sensible choice for patients whose symptoms are restricted to the nose and eyes. For therapy with oral antihistamines, newer antihistamines should be selected due to their better profile of side effects [26,27].

The objective of this noninterventional, observational study was to assess the effectiveness and safety of Ze 339 in patients with early allergic and late inflammatory symptoms of AR under conditions of daily practice. Apart from the primary diagnosis, the patients were not specifically selected, and the treatment was completely at the discretion of the responsible physician.

The efficacy and safety of Ze 339 for the treatment of seasonal AR have been demonstrated in several controlled clinical trials [14,15,21]. It could be demonstrated that Ze 339 is comparable to antihistamines in its efficacy and safety [15]. Most of the adverse events in these studies were of mild and moderate intensity across the treatment groups. The relative frequency of severe treatment-emergent adverse events was lower for *P. hybridus* film-coated tablets compared with placebo and fexofenadine and similar to the relative frequency in the cetirizine group [14]. Further, Ze 339 did not display the sedative side effects of common antihistamines [14,15]. It has been shown that Ze 339 exhibits a dual mode-of-action. Ze 339 exerts its antiallergic and anti-inflammatory effects through inhibition of leukotriene biosynthesis and proinflammatory mediators (e.g., LTB_4_, IL-8, histamine) in the early-phase response as well in the late-phase response and by the inhibition of intracellular calcium release. This has been confirmed by both preclinical and clinical studies [17,19,20].

Clearly, this study has some limitations. The study was explorative, and we tested many hypotheses without controlling the overall type 2 error rate, and all results should be interpreted accordingly. As this was an observational study, treatment indication, dosage, comedication and duration were heterogeneous. Further, a predefined control group was not included. However, patients participating in the present study could be divided into two distinct groups: 58.5% received on Visit 1 a monotherapy with Ze 339, and 41.5% received concomitant medication in addition to Ze 339. The comedication was composed of antihistamines (55.7%), glucocorticoids (26.8%), sympathomimetics (8.4%), leukotriene antagonists (0.8%) or combinations of these (4.4%). For the patients of the group receiving comedication, only the pharmacologically active drugs for allergic rhinitis and/or obstructive symptoms were considered for this study. Interestingly, patients receiving monotherapy showed a significantly lower allergic TSS (*p* = 0.023) and lower TSS variability (*p* = 0.001) at the end of treatment compared with those receiving concomitant medication. This result indicates that monotherapy was at least as effective as comedication.

The overall longer treatment duration may be explained by the high acceptance rate (93.9% at Visit 3) of treatment with Ze 339.

The current study did not reveal any safety concerns, and no signs of tolerance were observed. The range and incidence of adverse effects were very small, and the interaction profile with other medicinal products was favorable. Thus, Ze 339 is suitable for self-treatment of AR, as the patient is capable of monitoring the symptoms and the progress made without medical supervision. Furthermore, the product has a very low potential for abuse and has a well-characterized incidence of adverse events, which are typically mild.

In summary, the results of the present observational study not only confirmed the clinical effectiveness and safety of Ze 339 in nonselected patients with early allergic and late inflammatory symptoms of AR but also revealed new data about the average treatment duration and the preferred dose Ze 339 taken by patients (two tablets daily).

## 4. Materials and Methods

### 4.1. Study Design

The study was conducted as an open prospective noninterventional, observational study by 62 general practitioners and medical specialists (allergologists) in Switzerland. Patients were recruited during peak hay fever season in Switzerland (March–June in 2012 and 2013). Data were collected between March 2012 and October 2013.

### 4.2. Ethics

According to Swiss law, because the study was a noninterventional, observational study, no authorization by the Swiss Health Authority, Swissmedic, was required. However, due to its multicenter nature, the study was reviewed and approved by 11 independent local State Ethics Committees in Switzerland. All patients signed a written informed consent form for the use of their study-related data before participation. The responsible physicians were free in the choice of drug treatment and doses given. No additional diagnostic and therapeutic interventions to the standard of care were requested. No patient data, which could be used to identify the patients were recorded. The study complied with the STROBE requirements for cohort studies for strengthening the reporting of observational studies in epidemiology [28].

### 4.3. Study Medication

Ze 339, a CO_2_ extract (drug–extract ratio = 50–100:1) from the leaves of *Petasites hybridus* L., is registered for the treatment of hay fever (allergic rhinitis) symptoms and related symptoms in the eyes, nose and throat. The film-coated tablets contained 20–40 mg CO_2_ extract Ze 339 corresponding to 8 mg petasins. The batch of Ze 339 tested contained the following constituents: total petasins (in the investigated batch: petasin 18.9%, isopetasin 15.4%, and neopetasin 2.1%, respectively) and total fatty acids (34.0%). The remaining 29.6% contained other constituents (e.g., essential oils, sterols, minerals, and vitamins). An HPLC fingerprint of Ze 339 is provided in the Supplementary Materials (Figure S1).

Some *Petasites hybridus* extracts have been shown to contain some PAs [29]. In the manufacturing process of Ze 339, however, PAs are specifically removed so that the final extract contains only traces of PAs (below 2 ppb) as demonstrated by a highly sensitive UPLC TOF MS analytical method [11]. For this study, only commercially available medication was used.

### 4.4. Participants

#### 4.4.1. Inclusion Criteria

All patients who presented symptoms of acute allergic rhinitis and for whom drug therapy was intended qualified for inclusion in the study. Additionally, a signed informed consent form was mandatory.

#### 4.4.2. Exclusion Criteria

There were no special exclusion criteria, especially no restriction on concomitant health conditions, medication and treatments. Apart from the primary diagnosis, the patients were not selected, and the treatment was completely at the discretion of the responsible physician.

### 4.5. Outcome Measures

Data were collected during three documented visits per patient (see Figure 1). The intermediate visit (Visit 2) was suggested to be 2–4 weeks after the baseline visit (Visit 1). A final visit (Visit 3) was suggested to take place approximately 2–4 months after the intermediate visit (Visit 2). At screening and inclusion (Visit 1), demographic details and medical history were recorded. Assessment of the severity of AR, inflammatory and quality of life (QoL) symptoms was requested at all visits. Thirteen symptoms were recorded on a scale from 0 (not present) to 10 (unbearable). The symptoms can be classified into five antiallergic (rhinorrhea, nasal congestion, nasal itching, sneezing and eye itching), three anti-inflammatory (conjunctivitis, symptoms of sinusitis and obstructive airways disorder) symptoms, as well as five quality of life items (sleeping disorders, tiredness over the day, concentration difficulties, impairment of sportive activities and impairment of daily activities). The scale has been transformed into 4 categories: 0 = absent (corresponding to 0–1), 1 = mild (2–4), 2 = moderate (5–7) and 3 = severe (8–10), as suggested by the recent FDA guideline [30] for the study of antiallergic drugs. During the visits, adverse events (AEs), further treatment, concomitant symptoms of atopic diseases, medication, the patient and physician’s satisfaction with the treatment effectiveness and the physician’s satisfaction with the treatment tolerability were recorded by the investigator.

Demographic and safety parameters were assessed for each patient included in the study. All patients with fully documented Visit 1 and Visit 2 (regardless of whether they were being treated with Ze 339 or not) were included in the statistical analysis according to an intention-to-treat (ITT) approach. All patients with fully documented Visit 1 were included in the safety analysis. The most important variables of effectiveness and safety were analyzed comparing Visit 1 and Visit 2. Patients under treatment with Ze 339 until the final visit were analyzed for effectiveness (TSS) and safety.

### 4.6. Statistical Analysis

It was planned to include 300 patients in the study. This number was not based on a formal sample size estimation but on practical considerations and on the hay fever season. Descriptive statistical analysis of all recorded data was performed using IBM SPSS Statistics for Windows, Version 24.0, Armonk, NY, USA. Graphical analysis was performed using Origin 2018 Software, OriginLab Corp., Northampton, MA, USA. The exploratory descriptive analysis comprised the number of observations (n), mean, standard deviation (SD), median, minimum (Min) and maximum (Max). Differences in frequencies between study subgroups were analyzed by Chi-square or Fisher’s exact test, as appropriate. Changes in TSS from Visit 1 to Visit 2, Visit 2 to Visit 3 and Visit 1 to Visit 3 were analyzed by the two-sided nonparametric Wilcoxon Signed-Rank test. Differences in the variance of treatment effects were tested by Levene’s test. For repeated tests, *p*-values were adjusted for multiplicity of testing by use of Bonferroni’s correction. All values of *p* < 0.05 were considered significant.

## 5. Conclusions

In conclusion, the effectiveness of *P. hybridus* leaf extract Ze 339 for the treatment of early allergic and late inflammatory symptoms of allergic rhinitis could be confirmed in this non-interventional observational study. The study also revealed new data about the average treatment duration and the preferred dose Ze 339 taken by patients (two tablets daily).

## Figures and Tables

**Table 1:** Demographic data of the study population.

	Baseline Visit (*n* = 226)	Visit 2 (*n* = 205)	Final Visit (*n* = 131)
**Sex:**			
Male	90	83	44
Female	136	122	87
**Age (mean ± SD)**	37.3 ± 17.3	37.7 ± 17.5	37.1 ± 17.0
**Age distribution (years)**		Count (Percent)	
<12	1 (0.4%)	1 (0.5)	0
12–18	39 (17.3%)	35 (17.1%)	21 (16.0%)
19–35	74 (32.7%)	64 (31.2%)	43 (32.8%)
36–60	94 (41.6%)	87 (42.4%)	58 (44.3%)
>60	18 (8%)	18 (8.8%)	9 (5.9%)

**Table 2:** Baseline medical history (*n* = 226).

		*n*	Percent
Diagnosis	Seasonal AR	90	39.8
	Perennial AR (possibly including seasonal AR)	93	41.1
	unknown	43	19.0
Other symptoms	No other symptoms	128	56.6
of atopic disease	(Rhino-) Sinusitis	50	22.1
	(allergic) bronchial asthma	42	18.6
	Atopic dermatitis	30	13.3
	Other symptoms	19	8.4

## Data Availability

Data are available on request from the authors.

## Supplementary Materials

The following are available online at https://www.mdpi.com/1424-8247/14/3/180/s1, Figure S1: A HPLC fingerprint of Ze 339.
