# Supplementary material for: Treatment of Early Allergic and Late Inflammatory Symptoms of Allergic Rhinitis with Petasites Hybridus Leaf Extract (Ze 339): Results of a Noninterventional Observational Study in Switzerland
Source: Pharmaceuticals (Basel). 2021 Feb 24;14(3):180. doi: 10.3390/ph14030180 (PMC7996175; doi:10.3390/ph14030180)
Supplement: Supplementary file 1 [file pharmaceuticals-14-00180-s001.zip › pharmaceuticals-1106565-supplementary.pdf]

Supplemental material to:

Treatment of early allergic and late inflammatory symptoms of allergic rhinitis with Petasites hybridus leaf extract (Ze 339): Results of a non-interventional observational study in Switzerland

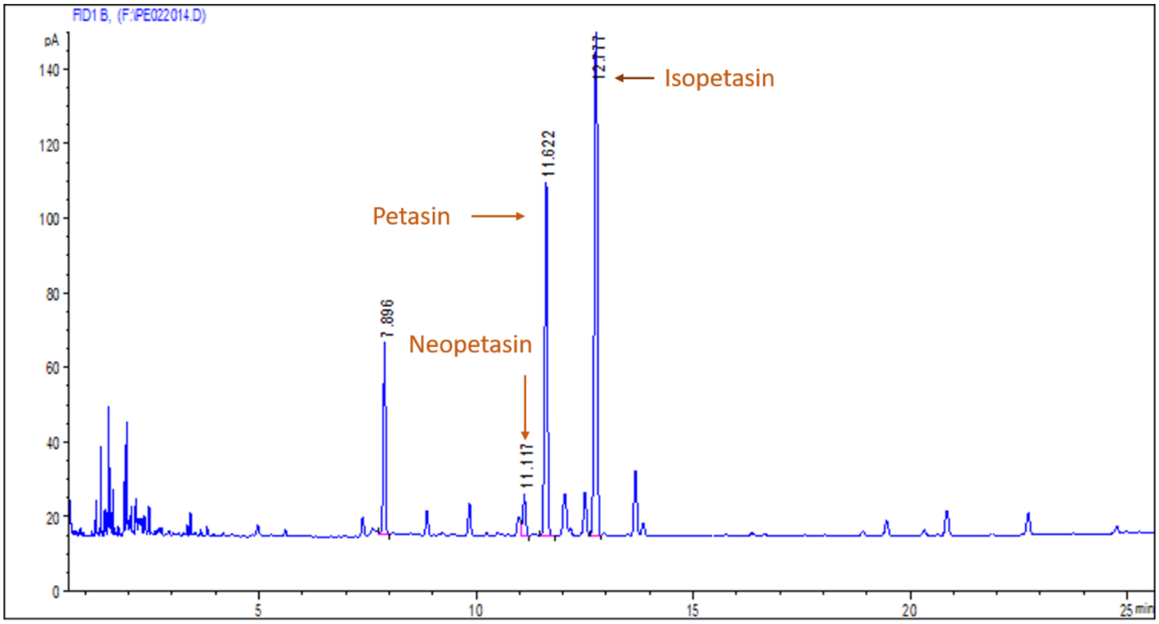

Figure S1: Gas chromatogram of Petasites hybridus leaf extract Ze 339 (batch 091376). Quantitative determination of total petasins (petasin, isopetasin, neopetasin), the active compounds of Ze 339 using gas chromatography and a flame ionization detector (FID). GC-column 100% polydimethylsiloxane (e.g. DB-1, length: 25 m, ID: 0.32 mm, dF: 0.52 µm); Injector temperature: 270 °C; Injection volume 1 µL.
